# Mining the Collaborative Networks: A Machine Learning-Based Approach to Firm Innovation in the Digital Transformation Era

**DOI:** 10.3390/e28030357

**Published:** 2026-03-22

**Authors:** Wenhao Zhou, Zhiwei Zhang

**Affiliations:** 1 Business School, Putian University, Putian 351100, China; 2 College of Business Administration, Capital University of Economics and Business, Beijing 100070, China

**Keywords:** firm innovation, digital transformation, collaborative network, machine learning, combined effect

## Abstract

Understanding how collaborative network structures and digital transformation jointly shape firm innovation has become a critical issue amid rapid technological change. Drawing on social network theory and a configurational perspective, this study investigates the nonlinear and interactive effects of collaborative network characteristics and digital transformation on firm innovation performance. Using patent data from Chinese listed manufacturing firms for the period between 2012 and 2022, inter-firm technological collaboration networks are constructed based on co-patenting relationships. A Classification and Regression Tree (CART) model is employed to uncover complex configurational patterns, complemented by regression-based robustness tests. The results reveal that innovation outcomes are not driven by single network attributes but by joint configurations of structural hole positions, centrality measures, and digital transformation. Among all factors, structural holes emerge as the most influential determinant. The findings further show that digital transformation interacts with network positions, generating multiple paths leading to high or low innovation performance. Model comparisons demonstrate that the CART approach outperforms traditional linear models in capturing nonlinear effects. This study contributes to the literature by highlighting the configurational logic of collaborative innovation and providing a machine learning-based framework for analyzing network–digital transformation interplay.

## 1. Introduction

The digital era has fundamentally reconfigured the landscape of industrial innovation, particularly within the manufacturing sector, where technological convergence and interconnected knowledge production have become central to competitive advantage [1]. Manufacturing firms worldwide are navigating a paradigm shift characterized by the integration of digital technologies into production processes, business models, and inter-organizational relationships [2]. This transformation, often encapsulated under the rubric of Industry 4.0, has profound implications for how firms innovate, collaborate, and compete [3]. The proliferation of digital technologies has simultaneously transformed how firms interact with external partners and how they leverage these interactions for innovation outcomes, creating both opportunities and challenges that require systematic investigation [4]. Understanding the mechanisms through which firms enhance their innovation performance in this new landscape is therefore of critical importance to both academic scholarship and managerial practice.

The theoretical foundations of innovation management have long emphasized that firms do not innovate in isolation but rather through complex networks of inter-organizational relationships [5]. Collaborative networks provide firms with access to diverse knowledge resources, facilitate resource sharing, enable risk mitigation in innovation activities, and create opportunities for synergistic value creation [6]. The structural properties of these networks, including network centrality, structural holes, and collaboration intensity, have been extensively documented as significant determinants of firms’ innovative output, as they shape the flow of information and the nature of knowledge recombination [7]. Firms occupying central positions in collaborative networks benefit from superior access to novel information and greater influence over network resources, while those spanning structural holes gain advantages from brokering non-redundant information flows [8]. The intensity and diversity of collaborative relationships further condition the extent to which firms can mobilize external knowledge for innovation purposes [9].

Concurrently, a substantial body of literature has emerged examining the transformative effects of digitalization on firm innovation [10]. Digital technologies enhance innovation performance through multiple mechanisms: they reduce information asymmetry between innovation partners, increase research and development efficiency through simulation and modeling capabilities, enable new forms of knowledge creation via data analytics, and facilitate the recombination of knowledge across previously distinct technological domains [11]. Firms undergoing digital transformation develop capabilities that fundamentally alter their innovation processes, from opportunity identification to knowledge integration and commercial exploitation. The extent to which firms embrace digital transformation has thus been identified as a key differentiator in their innovation outcomes [12].

Despite the richness of these two research streams, they have largely developed in parallel with limited theoretical integration. Studies examining collaborative networks have typically treated digital capabilities as control variables rather than as potential moderators of network effects, while research on digital transformation has often focused on firm-internal processes without systematic attention to firms’ external network positions [13]. This theoretical fragmentation is problematic because collaborative networks and digital transformation are not independent forces shaping innovation; rather, they are likely to interact in complex ways that single-lens approaches cannot capture [14]. The digital transformation context introduces additional complexity, digital capabilities may fundamentally alter how firms benefit from their network positions, potentially amplifying the returns to certain network configurations while attenuating the advantages of others [15].

A more fundamental limitation of the existing literature concerns the nature of the relationship between these two drivers of innovation. The prevailing analytical approach has been to examine the additive or linear interactive effects of network characteristics and digital transformation on innovation performance. This approach implicitly assumes that the influence of these factors is uniform across firms and that their joint effect can be adequately captured through conventional regression techniques [16]. However, such assumptions may be untenable given the complexity of the phenomena under investigation. The relationship between technology collaboration networks, digital transformation, and innovation performance is likely characterized by nonlinearities, asymmetries, and configurational dynamics that linear models cannot adequately represent. Different combinations of network positions and digital capabilities may be equally effective in producing high innovation performance, while the same configuration may yield different outcomes depending on unobserved contextual factors. These considerations suggest the need for analytical approaches that can accommodate complex interactions and reveal the multiple pathways through which firms achieve innovation success.

Recent studies in machine learning and innovation management offer promising avenues for addressing these limitations [17,18]. Decision tree-based methods, including classification and regression trees, random forests, and gradient boosting machines, are particularly well-suited for examining configurational effects in innovation research [19]. These techniques can automatically detect interactions among predictors, accommodate nonlinear relationships, and reveal the specific combinations of conditions that lead to high innovation performance. Unlike traditional regression approaches that require researchers to specify interaction terms a priori, machine learning methods allow the data to reveal complex interaction structures without strong parametric assumptions. These methods are increasingly being recognized in management research as valuable tools for theory development and for testing propositions about configurational causation [20].

Despite these advances, significant gaps remain in the literature. First, existing research has predominantly examined the main effects of either technology collaboration networks or digital transformation on innovation performance, leaving their joint effects largely unexplored. The question of how specific network positions and digital transformation capabilities combine to produce innovation outcomes remains unanswered, as does the question of whether multiple equally effective configurations exist. Second, studies that do consider interactions typically rely on linear moderation models that may miss the complex, nonlinear relationships that characterize these phenomena [21]. The assumption that the effect of network centrality is uniformly moderated by digital transformation across its entire range is unlikely to hold in practice, yet this assumption is embedded in conventional analytical approaches. Third, the manufacturing sector, where technology collaboration networks are particularly dense and digital transformation is especially consequential, has received insufficient attention in studies combining network and digitalization perspectives using machine learning-based analytical methods.

This study addresses these issues by investigating the configurational effects of technology collaboration network characteristics and digital transformation on firm innovation performance in the manufacturing sector. Drawing on social network theory and the resource-based view, and leveraging recent advances in machine learning, this research employs decision tree-based methods to identify the combinations of network positions and digital transformation that are associated with high innovation performance. Specifically, this study addresses two interrelated research questions. First, what configurations of technology collaboration network characteristics and digital transformation are associated with high firm innovation performance? Second, do multiple equally effective configurations exist, and if so, how do they differ in terms of the network and digital transformation conditions they comprise? By addressing these questions through a configurational lens and using decision tree methods, this study aims to provide a more complete and nuanced understanding of how firms enhance innovation performance in the digital era.

This investigation makes several contributions to the innovation management literature. First, it responds to calls for greater integration between network-based and digital-based explanations of firm innovation by developing a configurational framework that captures their joint effects [22]. Second, it advances understanding of the complex interplay between technology collaboration networks and digital transformation by moving beyond linear interaction models to examine nonlinear and configurational relationships. Third, it introduces decision tree-based machine learning methods to the study of innovation networks and digital transformation, demonstrating the value of these techniques for revealing complex patterns that traditional regression approaches may miss. Compared with traditional econometric approaches that primarily focus on net effects under linear assumptions, machine learning methods provide an alternative perspective for identifying nonlinear interactions and configurational patterns among multiple variables. In this study, the CART model is employed to uncover decision rules and heterogeneous paths leading to different innovation outcomes. Importantly, the machine learning results are further validated through regression-based robustness tests, which allows the study to combine the interpretability of configurational analysis with the statistical inference of conventional econometric models.

The remainder of this paper is organized as follows: Section 2 reviews the literature on technology collaboration networks, digital transformation, and firm innovation. Section 3 describes the research design, including data sources, variable measurement, and the methodology. Section 4 presents the data analysis and results, encompassing descriptive statistics, model construction and comparison, interpretation of decision rules, and robustness checks. Section 5 concludes with theoretical contributions, managerial implications, study limitations, and future research directions.

## 2. Literature Review

This section presents a comprehensive review of the literature on technology collaboration networks and digital transformation, examining their respective relationships with firm innovation performance. Section 2.1 discusses collaborative networks and innovation. Section 2.2 addresses digital transformation and innovation. This section also develops the theoretical framework for examining their combined configurational effects.

### 2.1. Collaborative Network and Firm Innovation

The role of inter-organizational relationships in shaping innovation outcomes has been a central concern in management research for decades [23]. Collaborative networks provide firms with access to diverse knowledge resources, facilitate resource sharing, and enable risk mitigation in innovation activities [24]. Within the manufacturing sector, technology-oriented collaborations have emerged as particularly critical mechanisms for knowledge acquisition and recombination, as technological complexity increasingly exceeds the boundaries of individual firms [25].

The structural properties of technology collaboration networks have been extensively documented as significant determinants of firms’ innovative output. Network centrality, reflecting a firm’s prominence and connectivity within the collaborative structure, enhances innovation performance by providing superior access to novel information and greater influence over network resources [26]. Firms occupying central positions benefit from heightened awareness of technological developments and increased opportunities for knowledge exchange with diverse partners [27]. Conversely, structural holes between non-redundant contacts in a network offer brokerage advantages that enable firms to access diverse information flows and combine knowledge from disparate technological domains [28]. The intensity of collaborative relationships, measured through repeated interactions and the depth of engagement, further conditions the extent to which firms can mobilize external knowledge for innovation purposes, with deeper collaborations facilitating tacit knowledge transfer and trust development [29,30].

Empirical evidence from manufacturing contexts confirms that firms strategically position themselves within technology collaboration networks to enhance innovation capabilities [31]. However, the innovation returns to particular network positions are not uniform; they depend on firm-specific factors including absorptive capacity, technological endowment, and strategic orientation [32]. This contingency perspective suggests that the value of network embeddedness is conditional on internal firm characteristics, an insight that motivates the examination of interactions between network positions and firm-level digital capabilities.

### 2.2. Digital Environment

Concurrent with developments in network research, substantial studies have begun to explore the transformative effects of digitalization on firm innovation [33]. Digital transformation refers to the integration of digital technologies into organizational processes, business models, and value creation activities, fundamentally altering how firms operate and compete [34]. Digital technologies reduce information asymmetry between innovation partners by enabling real-time data sharing and transparent communication. They increase research and development efficiency through simulation, modeling, and virtual prototyping capabilities that accelerate experimentation and reduce costly physical trials. Digital technologies enable new forms of knowledge creation via advanced data analytics, revealing patterns and relationships that would otherwise remain undetected [35]. Furthermore, digitalization facilitates the recombination of knowledge across previously distinct technological domains by providing common platforms and interoperability standards that bridge disciplinary boundaries.

The extent to which firms embrace digital transformation has been identified as a key differentiator in innovation outcomes [36]. Firms developing advanced digital capabilities demonstrate superior ability to identify innovation opportunities, integrate diverse knowledge inputs, and accelerate the commercialization of new products and processes. However, research also indicates that digital transformation entails significant organizational challenges, including capability gaps, cultural resistance, and coordination complexities that can undermine innovation performance if not effectively managed [37]. These findings underscore the need to understand the conditions under which digital transformation enhances rather than impedes innovation. When firms simultaneously navigate network relationships and digital transformation trajectories, the innovation consequences cannot be reliably predicted from the sum of their separate effects [38]. The relationship between networks, digitalization, and innovation may exhibit nonlinearities and threshold effects, where the benefits of combining these factors only materialize beyond certain levels of each.

The uncertainty inherent in the joint effects of networks and digitalization reflects broader characteristics of innovation systems in the digital era. Innovation processes increasingly involve complex interactions across technological domains, organizational boundaries, and knowledge types, generating emergent outcomes that resist simple linear explanation. The proliferation of digital technologies introduces additional layers of complexity, enabling new forms of connectivity while simultaneously creating novel sources of unpredictability. Within this context, firms face considerable ambiguity regarding how to configure their network relationships and digital investments to optimize innovation performance, as multiple configurations may prove effective and the optimal configuration may depend on firm-specific and contextual factors.

The preceding review suggests that understanding the joint effects of technology collaboration networks and digital transformation on firm innovation requires analytical approaches capable of capturing complex interactions, nonlinear relationships, and equifinal outcomes. Traditional regression-based methods, while valuable for testing linear moderation effects, impose assumptions that may not hold in this context.

**Figure 1:**
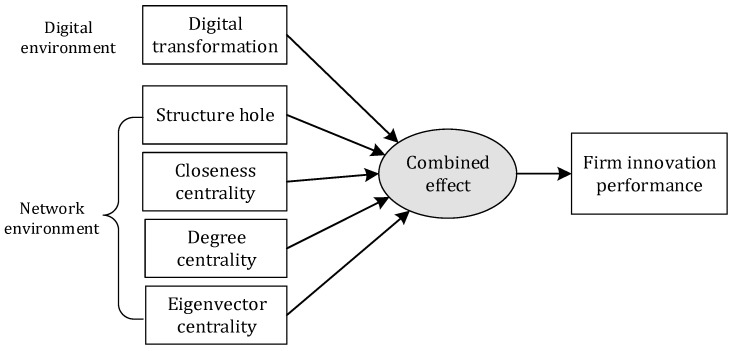
The combined effect framework of this study.

This study adopts a configurational perspective grounded in recognizing that technology collaboration network characteristics and digital transformation intensity combine in multiple ways to produce innovation outcomes. The configurational approach posits that firms achieve high innovation performance through different combinations of conditions, and that the effects of individual factors depend on their broader context [24]. Within this framework, technology collaboration network characteristics including centrality, structural holes, and collaboration intensity, and digital transformation intensity are viewed as interconnected elements whose combined configuration, rather than isolated effects, determines innovation outcomes. In light of this, a comprehensive framework is meticulously constructed as shown in Figure 1 to investigate the combined effect.

## 3. Research Design

This section describes the research design employed in this study. Section 3.1 presents the data sources and sample selection. Section 3.2 defines the measurement of key variables. Section 3.3 introduces the methods used in this study.

### 3.1. Data and Source

This study constructs a panel dataset of Chinese listed manufacturing firms by integrating patent data, collaborative network data, digital transformation indicators, and firm-level financial information. The core patent data are obtained from the Incopat database (https://www.incopat.com/, accessed on 11 June 2025), which provides comprehensive and structured information on patent applications in China. The research focuses on listed firms in the manufacturing sector. This choice is motivated by two considerations. First, manufacturing is one of the most active sectors in terms of digital transformation and technological upgrading in China, where the integration of digital technologies and industrial production is particularly prominent. Second, manufacturing firms rely heavily on inter-organizational technological collaboration to overcome complex innovation challenges, making them an appropriate context for examining collaborative networks and firm innovation in the digital era. Using the full list of Chinese A-share listed manufacturing firms as the sample frame, we match firm names with patent applicants in the Incopat database and collect all invention patent applications. A total of 180,607 patent records are obtained. For each patent, fields such as patent application number, application year, publication number, IPC classification codes, forward citations, co-applicant information, applicant name are retrieved.

To construct the collaborative network, joint patent applications are used to capture technological cooperation between firms. If two or more firms jointly apply for a patent, a collaborative tie is established between them. Considering the time-lag effect of innovation and to ensure sufficient network density [39], a three-year moving window is adopted to build the technological collaboration network. Specifically, networks are constructed for the periods 2010–2012, 2011–2013, and so forth, ultimately covering the observation window from 2012 to 2022. Based on these networks, structural indicators such as degree centrality, closeness centrality, eigenvector centrality, structural holes, and clustering coefficient are calculated for each firm-year observation.

To mitigate potential endogeneity concerns, all independent variables are lagged by one period relative to the dependent variable. Firm-level financial data and control variables are obtained from the CSMAR database. These variables include firm size, firm age, leverage, profitability and other basic characteristics. After merging the patent data, network indicators, digital transformation measures, and financial data, an unbalanced panel dataset of Chinese listed manufacturing firms for the period 2012–2022 is constructed.

### 3.2. Variables

#### 3.2.1. Firm Innovation Performance

Firm innovation performance (*FIP*) is measured using the forward three-year citations of firm patents, which reflect the technological impact and knowledge diffusion of a firm’s innovation output. Compared with simple patent counts, citation-based indicators capture the quality and influence of innovation and have been widely adopted in innovation research.

Specifically, this study uses the natural logarithm of the forward three-year citations of a firm’s granted patents to measure innovation performance. The logarithmic transformation helps reduce skewness and mitigate the influence of extreme values. To ensure the temporal order between explanatory variables and the dependent variable, the innovation performance variable is measured at time t, while all independent variables are lagged by one period.

#### 3.2.2. Digital Transformation

Digital transformation (*DT*) reflects the extent to which firms adopt and integrate digital technologies into their production, operation, and innovation processes. To capture this construct at the firm level, this study adopts a text mining approach based on annual reports.

Specifically, the annual reports of all Chinese A-share listed firms from 2012 to 2023 are collected and converted into machine-readable TXT format using Python 3.12.3. Annual reports provide detailed and standardized disclosures of firms’ strategic orientation, technological deployment, and digital investment, and therefore serve as an appropriate data source for measuring digital transformation.

To identify digital-related content, a domain-specific dictionary of digital transformation is developed. The dictionary contains 127 keywords, covering key digital technologies and application scenarios, including artificial intelligence, image recognition, intelligent data analysis, intelligent robotics, machine learning, deep learning, semantic search, biometric identification, facial recognition, speech recognition, identity authentication, autonomous driving, and natural language processing.

Based on this dictionary, the frequency of digital keywords in each firm-year annual report is calculated. To reduce the influence of document length and capture the relative importance of digital content, the term frequency–inverse document frequency (TF–IDF) method is employed to construct the digital transformation index.

The index is calculated as follows:

(1) $$DT_{i,t}=\sum_{w}\left[\ln(TF_{i,t,w}+1)\times \ln\left(\frac{N_{t}}{n_{t,w}+1}\right)\right]$$

where *TF_i_*_,*t*,*w*_ denotes the term frequency of digital keyword *w* in the annual report of firm *i* in year *t*; *N_t_* represents the total number of annual reports in year *t*; *n_t_*_,*w*_ indicates the number of annual reports in year *t* that contain keyword *w*. The logarithmic transformation is applied to mitigate the impact of extreme values. A higher value of the index indicates a higher level of digital transformation.

#### 3.2.3. Network Characteristics

The inter-firm technological collaboration network is constructed based on joint patent applications within a three-year moving window. Firms are treated as nodes and co-patenting relationships as undirected edges. This network structure reflects the channels through which firms access external knowledge, recombine technological resources, and coordinate innovation activities.

To capture firms’ heterogeneous structural positions in the collaborative innovation system, four network indicators are selected: degree centrality, closeness centrality, eigenvector centrality, and structural holes. These indicators respectively represent firms’ direct connectivity, knowledge access efficiency, embeddedness in the network core, and brokerage advantages [39]. Together, they provide a multidimensional characterization of the collaborative network environment.

Degree centrality (*DC*) measures the number of direct collaborative partners of a focal firm. It is calculated as

(2) $$DC_{i}=\frac{\sum_{j=1}^{n}a_{ij}}{n-1}$$

where *a_ij_ =* 1 if firm *i* and firm *j* jointly apply for a patent, and it equals 0 otherwise. In the technological collaboration network, degree centrality reflects the breadth of a firm’s external knowledge sources. Firms with higher degree centrality maintain more direct collaborative ties and are therefore exposed to more diverse technological information and complementary resources. Degree centrality is included because it captures the knowledge acquisition capacity derived from collaboration breadth, which is a fundamental mechanism through which networks influence innovation.

Closeness centrality (*CC*) signifies the degree to which a focal firm’s location assumes a central position within the network. A reduced shortest distances between the focal firm and other nodes in the immediate network corresponds to an elevated closeness centrality. The mathematical expression for this measure is provided as follows:

(3) $$CC\left(i\right)=\frac{1}{\sum_{i\neq j,j=1}^{N}d_{ij}}$$

where $d_{ij}$ denotes the shortest path length of node *i* to another nodes *j* in the network.

Structural holes (*SH*) measure the extent to which a firm bridges otherwise disconnected partners and are calculated as

(4) $$SH_{i}=1-\sum_{j}{\left(p_{ij}+\sum_{q}p_{iq}p_{qj}\right)}^{2}$$

where $p_{ij}$ represents the proportion of firm *i*’s network time and resources invested in the relationship with firm *j*. In the technological collaboration network, occupying structural holes provides brokerage advantages. Such firms can access non-redundant knowledge, control information flows, and recombine heterogeneous technologies.

Structural holes are included because they capture the knowledge recombination capability and brokerage power, which are key drivers of exploratory innovation.

Eigenvector centrality (*EC*) reflects the importance of adjacent nodes linked to the enterprise. Partners’ network structure has been termed second-order social capital [27]. When partners exhibit high centrality, the focal node itself is also more inclined to attract attention from others and is predisposed to forge connections. Referring to previous study [40], the eigenvector centrality of a node is proportional to the centrality of its connected nodes, adhering to the following equation:

(5) $$\lambda x_{i}=\sum_{j=1}^{n}a_{ij}x_{j},i=1,2,3,\ldots ,n$$

In particular, *n* denotes the number of firms in the network. *a_ij_* denotes the connection between two different nodes *i* and *j*. If these is a linkage between these two nodal firms, *a_ij_* = 1; otherwise *a_ij_* = 0. *λ* is the eigenvalue value of the adjacency matrix.

### 3.3. Methods

This study employs a two-stage analytical framework: Social network analysis followed by a machine learning classification model. First, social network analysis is used to construct the inter-firm technological collaboration network and to extract structural indicators that characterize each firm’s position in the knowledge collaboration system. Based on joint patent applications, an undirected network is built for each three-year window. Firms are represented as nodes and collaborative ties as edges. Network metrics are then calculated to transform relational data into quantitative variables that can be incorporated into the predictive model.

To examine the nonlinear and configurational effects of network characteristics and digital transformation on firm innovation performance, a Classification and Regression Tree (CART) model is employed. The CART algorithm recursively partitions the feature space into a set of homogeneous subgroups. At each node, all possible splits are evaluated and the split that produces the greatest reduction in impurity is selected.

Within the CART framework, this uncertainty is quantified through node impurity. A node is considered pure when most observations belong to the same innovation-performance category and impure when different categories are mixed. For a node *t*, its *Gini* impurity is defined as

(6) $$I_{G}(t)=1-\sum_{i=1}^{C}{\left[p(i|t)\right]}^{2}$$

where *C* denotes the number of classes. *p*(*i|t*) represents the proportion of observations in node *t* that belong to class *i*. A lower *Gini* value indicates that firms within the node share a similar innovation outcome and therefore exhibit a clearer configurational pattern. A split *s* divides the node into left and right child nodes *t_L_* and *t_R_*. The quality of the split is measured by the reduction in impurity, or *Gini* gain:

(7) $$\Delta I_{G}(s,t)=I_{G}(t)-\left(\frac{n_{L}}{n}I_{G}(t_{L})+\frac{n_{R}}{n}I_{G}(t_{R})\right)$$

where *n*, *n_L_*, and *n_R_* denote the sample sizes in the parent and child nodes. The algorithm selects the split that maximizes *ΔI_G_*(*s*,*t*), effectively identifying the feature and threshold that best separate different innovation types.

As this splitting rule is information-theoretic in nature, minimizing impurity is equivalent to increasing the predictability of the outcome. In information theory terms, it corresponds to maximizing information gain, the reduction in Shannon entropy *H*(*t*) after a split, where entropy is defined as

(8) $$H(t)=-\sum_{i=1}^{C}p(i|t)\log_{2}p(i|t)$$

While the *Gini* impurity is used for computational efficiency, it shares a consistent objective with entropy-based criteria: to partition the feature space by progressively reducing uncertainty in the classification of innovation types. In this study, the entropy-based splitting criterion is not only a computational tool but also reflects the uncertainty inherent in collaborative innovation systems. This configurational uncertainty can be interpreted as a form of system entropy, where the distribution of innovation outcomes across firms reflects the diversity and complexity of collaborative structures. Therefore, the entropy reduction achieved through node splitting represents the process of identifying more homogeneous configurational patterns within the innovation system.

## 4. Data Analysis and Results

In this section, the data analysis and empirical results are reported. Section 4.1 presents descriptive statistics and correlation tests. Section 4.2 describes the construction of the decision tree model. Section 4.3 provides model comparison results. Section 4.4 interprets the decision rules derived from the analysis. Section 4.5 reports robustness checks.

### 4.1. Descriptive Statistics and Correlation Test

Table 1 reports the descriptive statistics and Pearson correlation coefficients for the variables used in the empirical analysis. The sample consists of 1292 firm-year observations. In terms of the dependent variable, firm innovation performance (*FIP*) exhibits substantial variation across firms, with a mean value of 22.00 and a standard deviation of 21.93. The minimum value is 0, while the maximum reaches 138.1, indicating a highly skewed distribution in patent citation outcomes. This dispersion suggests pronounced heterogeneity in innovation performance among manufacturing firms, which is consistent with the uneven distribution of innovative outputs observed in prior studies.

Regarding the key explanatory variable, digital transformation (*DT*) shows a relatively small mean value (0.001) and a limited range (0, 0.015), reflecting the fact that digital transformation is still at an evolving stage among Chinese manufacturing listed firms during the observation period. Nevertheless, the positive and significant correlation between DT and FIP (r = 0.265, *p* < 0.001) provides preliminary evidence that digitalization is associated with improved innovation outcomes. For the network characteristics, degree centrality (*DC*) has a mean of 0.006 and is positively correlated with *FIP* (r = 0.418, *p* < 0.001), indicating that firms occupying more central positions in the collaborative network tend to achieve higher innovation performance. Closeness centrality (*CC*) has a mean of 1.186 but shows no significant correlation with *FIP*, suggesting that the efficiency of reaching other actors in the network does not directly translate into higher innovation output at the bivariate level. Structural holes (*SH*) display a positive and significant relationship with *FIP* (r = 0.352, *p* < 0.001), implying that firms bridging disconnected partners benefit from access to heterogeneous knowledge and non-redundant information. Eigenvector centrality (*EC*), however, is negatively correlated with *FIP* at the 5% significance level (r = −0.064, *p* < 0.05), indicating that being connected to highly central partners does not necessarily lead to superior innovation performance and may even generate constraints due to network redundancy or excessive embeddedness.

The correlations among the independent variables are generally within an acceptable range. Degree centrality is moderately correlated with structural holes (r = 0.541, *p* < 0.001), which is expected because firms with more collaborative ties are more likely to span structural gaps. Closeness centrality is strongly correlated with eigenvector centrality (r = 0.671, *p* < 0.001), reflecting their shared dependence on the overall network structure. However, none of the correlation coefficients exceeds the commonly accepted threshold that would indicate severe multicollinearity. Therefore, the variables are suitable for subsequent machine learning analysis.

Overall, the descriptive statistics reveal significant heterogeneity in both innovation performance and network positions, while the correlation results provide initial support for the role of collaborative network structures and digital transformation in shaping firm innovation.

### 4.2. Construction of Decision Tree Model

To uncover the configurational and nonlinear effects of collaborative network characteristics and digital transformation on firm innovation performance, the CART model was constructed. Unlike traditional regression approaches that focus on net effects, the CART algorithm identifies hierarchical splitting rules and interaction structures among explanatory variables, thereby revealing multiple equivalent causal paths leading to high innovation performance.

Specially, to reflect the structural heterogeneity in innovation outcomes and to make the classification results directly interpretable in terms of innovation patterns, *FIP* was transformed into a binary variable. Specifically, the mean value of *FIP* for the full sample was used as the threshold. Observations with *FIP* values greater than the sample mean were coded as 1 (high innovation performance), while those below the mean were coded as 0 (low innovation performance). After the transformation, the number of firms in the high-innovation group is 629, while the low-innovation group contains 663 observations. The relatively balanced distribution between the two classes ensures that the classification model does not suffer from severe class imbalance and improves the reliability of the CART estimation and the interpretability of the extracted decision rules.

The full dataset was randomly divided into a training set and a test set with a ratio of 0.75:0.25. The training set was used for model estimation and hyperparameter optimization, while the test set was employed for out-of-sample evaluation. Such a hold-out validation strategy ensures that the predictive performance reflects the generalization ability of the model rather than in-sample fit. To obtain a robust tree structure and avoid model overfitting, hyperparameter tuning was conducted using Grid-Search-CV combined with 10-fold cross-validation. In this procedure, the training set was partitioned into ten mutually exclusive subsets; nine subsets were used for model training and the remaining subset for validation, and the process was repeated iteratively. The average performance across the ten folds was used as the evaluation criterion. The area under the ROC curve (ROC-AUC) was selected as the scoring metric during parameter optimization. Compared with the accuracy, the AUC is threshold-independent and captures the model’s ability to discriminate between high- and low-innovation firms across different classification cutoffs, making it more suitable for innovation studies where class distributions may be uneven.

The parameter grid included key complexity-control parameters including maximum tree depth (max_depth), which determines the number of hierarchical splits; the minimum number of samples required to split an internal node (min_samples_split); the minimum number of samples required to form a leaf node (min_samples_leaf); and the cost–complexity pruning parameter. The optimal parameter combination was selected based on the highest cross-validated AUC value.

**Figure 2:**
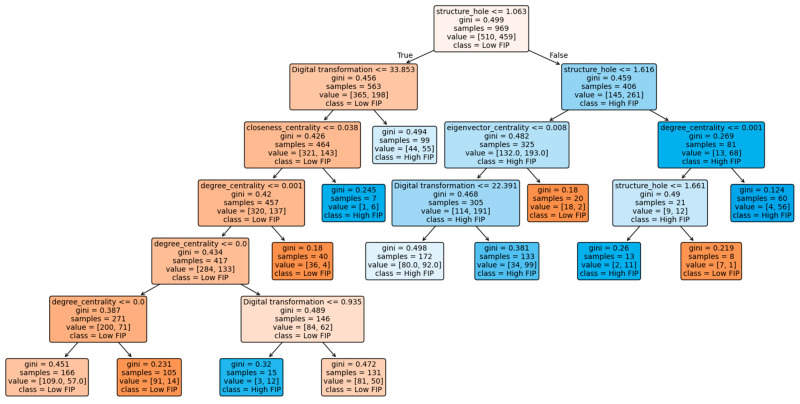
The CART decision tree of firm innovation performance.

The optimized CART model produces a tree with a depth of 6, 25 total nodes, and 13 leaf nodes, as shown in Figure 2. This structure indicates that the model captures sufficiently complex interaction patterns while maintaining an interpretable hierarchical form.

### 4.3. Model Comparison

To evaluate the predictive validity and methodological contribution of the CART model, its performance was compared with three widely used benchmark models, namely logistic regression, random forest, and support vector machine (SVM). These models represent different analytical logics: logistic regression captures linear net effects, random forest reflects ensemble-based nonlinear learning, and SVM provides a nonlinear classification framework with strong generalization ability but limited interpretability.

Table 2 reports the predictive performance of the four models on the test set. Overall, the CART model achieves the highest AUC value (0.718), indicating the strongest ability to distinguish between high and low innovation performance firms. In terms of classification accuracy, the random forest slightly outperforms the CART model (0.669 and 0.659), but its AUC is lower than that of the CART model. Since AUC is threshold-independent and better captures the overall discriminative power of the model, this result suggests that the decision tree provides more reliable classification across different cutoff values.

Regarding precision and recall, the models exhibit different predictive tendencies. The SVM model yields the highest precision (0.746), indicating a conservative classification strategy that reduces false positives but at the cost of a relatively low recall (0.518). Logistic regression also shows a low recall (0.524), implying limited capability in identifying firms with high innovation performance. In contrast, the CART model achieves the highest recall (0.706), demonstrating a stronger ability to correctly identify high-innovation firms, which is particularly important for innovation management research that focuses on the structural conditions leading to superior innovation outcomes.

**Figure 3:**
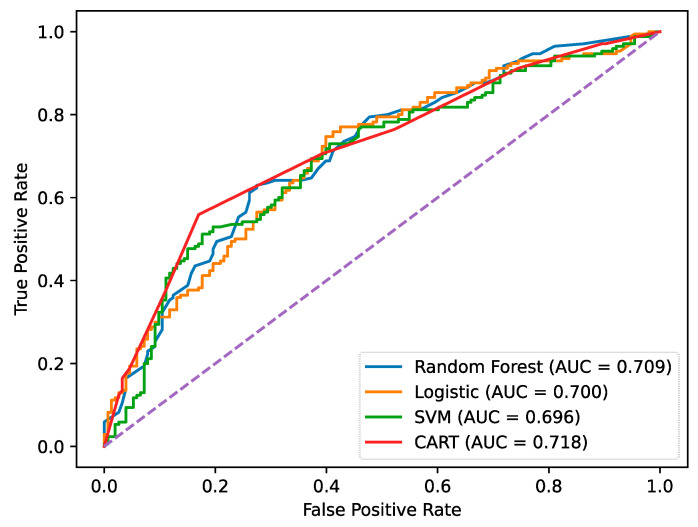
ROC curves of diverse models.

More importantly, although random forest shows competitive performance in terms of accuracy, it operates as a black-box ensemble model and cannot provide explicit configurational paths. The logistic regression model is restricted to linear and additive effects, and the interaction-based mechanisms must be specified ex ante. The SVM model, while nonlinear, also lacks interpretability in terms of hierarchical decision logic. By comparison, the CART model not only achieves the best overall discriminative performance in terms of AUC but also generates transparent decision rules shown in Figure 2 that reveal how collaborative network positions and digital transformation jointly shape innovation performance through multiple equivalent configurations. Receiver operating characteristic (ROC) curves of these models are demonstrated in Figure 3.

Therefore, the empirical results suggest that the nonlinear and configurational relationships identified in this study are not artifacts of a specific modeling technique. Instead, they represent stable structural patterns that remain observable when compared with both linear and advanced machine learning models. This finding justifies the use of the CART approach as an appropriate analytical tool for uncovering the synergistic and contingent effects of collaborative networks in the digital era.

### 4.4. Interpretation of Decision Rules

To enhance the robustness and interpretability of the extracted classification structure and to avoid overfitting caused by overly specific terminal nodes, two additional evaluation criteria, *Support* and *Confidence*, are introduced to further prune the decision rules. *Support* refers to the proportion of observations in the total sample that satisfy a given rule, while *Confidence* represents the proportion of correctly classified observations among those that meet the rule conditions. For the specific decision rule A→B, its Support and Confidence are as follows:

(9) $$Support\left(A\to B\right)=\frac{\delta \left(A\cap B\right)}{T}\times 100\%$$

(10) $$Confidence\left(A\to B\right)=\frac{\delta \left(A\cap B\right)}{\delta \left(A\right)}\times 100\%$$

where *T* is the total number of samples and $\delta \left(A\right)$ represents the proportion of samples that satisfy the sample set. The higher its *Support* and *Confidence*, the more convincing it is, with a maximum value of 1. Decision makers can achieve significant rule discovery and excavation by setting thresholds for evaluation criteria in advance, similar to the significance levels in traditional regression analysis.

After pruning based on these criteria, six key decision rules were retained, as reported in Table 3. These rules reveal multiple configurational paths leading to either high or low firm innovation performance, indicating that innovation outcomes are not determined by a single network attribute but by the joint effects of digital transformation and firms’ structural positions in collaborative networks.

A closer examination of the extracted rules shows clear structural regularities across different innovation states. The first group of rules corresponds to low innovation performance. The dominant rule in this category has the highest support (47.16%) and a confidence level of 70.02%, suggesting a highly representative structural pattern in the sample. This configuration is characterized by a low level of digital transformation combined with weak network embeddedness, reflected in low degree centrality, limited structural hole advantages, and low eigenvector centrality. This result indicates that firms lacking both digital capability and advantageous network positions face significant constraints in accessing heterogeneous knowledge and mobilizing external innovation resources.

Another low-innovation configuration, although with smaller support, also reflects a similar structural logic: firms located in peripheral network positions and without sufficient digital transformation are more likely to exhibit inferior innovation performance. These findings suggest that digital transformation alone cannot compensate for structural disadvantages when firms remain disconnected from the core of collaborative networks.

In contrast, several configurations are associated with high innovation performance. A representative path (*Support* = 31.48%, *Confidence* = 62.62%) shows that firms occupying advantageous structural hole positions can achieve superior innovation outcomes even without the highest levels of digital transformation. This highlights the critical role of brokerage positions in facilitating access to diverse knowledge pools and promoting recombinatory innovation.

The fifth high-innovation rule exhibits an extremely high confidence level (90.00%) despite relatively small support. This configuration indicates that when firms simultaneously possess strong structural hole advantages and higher network centrality, the probability of achieving high innovation performance increases substantially. Such firms act as key intermediaries in the collaborative network and are able to integrate dispersed knowledge resources efficiently.

Overall, these rules demonstrate three important empirical regularities. First, innovation performance is the outcome of configurational effects rather than the independent influence of single variables. Second, structural hole positions appear repeatedly in high-innovation configurations, indicating their dominant role in differentiating innovation outcomes. Third, digital transformation does not operate as a universally sufficient condition; instead, its effect varies across different network structural contexts. These findings provide a micro-level structural explanation for the heterogeneous innovation outcomes observed in collaborative networks. Rather than exerting a uniform net effect, digital transformation and network embeddedness jointly shape firms’ innovation performance through multiple equivalent configurations. This configurational logic helps reconcile the inconsistent results reported in previous regression-based studies and highlights the necessity of moving beyond linear analytical frameworks when examining innovation in the digital era.

### 4.5. Robustness Check

#### 4.5.1. Alternative Training–Testing Split Ratio

To examine whether the empirical results are sensitive to the data partitioning strategy, the baseline training–testing split ratio 0.75:0.25 was replaced with an alternative ratio of 0.8:0.2, while keeping all other model settings unchanged, including the parameter tuning procedure, pruning criterion, and evaluation metrics.

**Figure 4:**
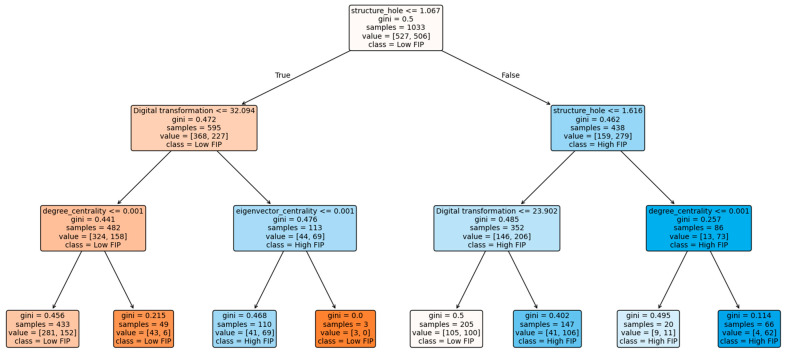
Robustness test results of replacement sample division.

The re-estimated decision tree obtained from the new training set exhibits a highly similar structure to the baseline model shown in Figure 4. In particular, structural hole remains the primary splitting variable at the upper levels of the tree, indicating its dominant role in differentiating firm innovation performance. The relative positions of digital transformation and the centrality-related indicators in the tree are also largely consistent with the baseline results. More importantly, the configurational patterns identified in the alternative specification continue to show that firm innovation performance is not determined by a single network attribute. Instead, it is jointly shaped by the interaction between firms’ structural positions in collaborative networks and their level of digital transformation. The coexistence of multiple classification paths leading to both high and low innovation performance further confirms the combinational and nonlinear nature of the relationship.

Overall, the similarity in tree topology, core splitting variables, and decision logic suggests that the main findings are not driven by a specific training–testing split and that the identified structural mechanisms are stable across different sample partitioning schemes.

#### 4.5.2. Regression-Based Check

To further validate the reliability of the decision-tree findings, a regression-based robustness test was conducted. This approach serves two purposes. First, it examines whether the core relationships identified by the machine learning model remain statistically significant under a parametric framework. Second, it tests whether the configurational effect extracted from the CART model can be supported by traditional econometric analysis.

Table 4 reports the OLS and Logit estimation results using firm innovation performance (*FIP*) as the dependent variable. Models (1) and (2) employ the continuous measure of *FIP*, while Models (3) and (4) use the binary innovation-performance variable. Model (1) and Model (3) include only the core explanatory variables, whereas Model (2) and Model (4) additionally control for firm-level characteristics.

The estimation shows that *DC*, *DT*, and *SH* remain significantly positive across most specifications, whereas *EC* exhibits a significantly negative effect. *CC* does not show stable significance. More importantly, the direction and significance of these variables are highly consistent with the primary splitting variables identified in the CART model, indicating that the dominant role of structural holes and the enabling effect of digital transformation are not model-specific outcomes but stable empirical relationships.

In the second stage, the most representative decision rule with the largest sample coverage identified by the CART model is transformed into a configurational dummy variable:

(11) $$Configuration1=\left(DT\leq 33.853\;\&\;CC\leq 0.038\;\&\;SH\leq 1.063\right)$$

This variable equals 1 when a firm falls into this specific configurational condition and equals 0 otherwise. The newly constructed configuration variable is then introduced into the regression model together with the original network indicators. The regression results are shown in Table 5.

The coefficient of Configuration1 is significantly negative across all model specifications. This indicates that firms located in this specific low *DT*–low *CC*–low *SH* configuration are significantly associated with lower innovation performance. After the configuration variable is included, the explanatory power of the models further increases, and the core variables largely retain their significance and direction, demonstrating that the configurational condition captures additional structural information beyond individual network indicators.

Overall, the results reported in Table 4 and Table 5 provide strong and consistent support for the robustness of the main findings. The baseline regressions confirm that the direction and significance of the core network variables and digital transformation are highly consistent with the variable importance and splitting structure identified by the CART model. Meanwhile, the configurational condition extracted from the decision tree remains statistically significant after being transformed into a regression-based dummy variable and after the benchmark control variables is simultaneously introduced into the model.

## 5. Conclusions and Discussion

In this section, the main conclusions of the study are summarized, followed by a discussion of managerial implications. The limitations of the research are acknowledged, and directions for future inquiry are proposed.

### 5.1. Conclusions

This study investigates how digital transformation and firms’ network structural positions jointly shape firm innovation performance from a configurational perspective. By integrating social network analysis with a CART decision tree model, this research moves beyond the traditional net-effect paradigm and uncovers multiple causal paths leading to heterogeneous innovation outcomes.

First, the empirical results reveal that firm innovation performance is not driven by a single factor but by the synergistic effects of digital transformation and network structural attributes. The decision tree structure shows that structural holes play a dominant role in differentiating innovation performance, indicating that occupying brokerage positions in collaborative networks constitutes a critical condition for innovation advantage. Meanwhile, digital transformation does not function as an independent determinant; instead, it interacts with firms’ structural positions and other network characteristics to form multiple configurational paths. This finding highlights the non-linear and combinational nature of the digital transformation–innovation relationship.

Second, the extracted decision rules identify several typical pathways leading to high and low innovation performance. Firms achieving high innovation performance tend to be embedded in network structures characterized by advantageous brokerage positions and specific relational conditions, whereas low innovation performance is more likely to occur when firms simultaneously exhibit weak digital transformation and disadvantaged network locations. These results indicate that different combinations of conditions may produce similar innovation outcomes, thereby providing empirical evidence for the equifinality of firm innovation performance. Meanwhile, the model comparison demonstrates that the CART model outperforms logistic regression and SVM in recall and AUC, and exhibits comparable predictive power to the random forest model while maintaining superior interpretability. This study therefore demonstrates how machine learning approaches can complement rather than replace traditional econometric methods in innovation research.

Third, the results consistently identify structural holes as the most critical factor differentiating innovation performance. As the root node of the decision tree and the primary splitting condition in multiple decision rules, structural holes determine the overall direction of the classification and define the basic boundary between high and low innovation performance. This finding indicates that firms occupying brokerage positions in technological collaboration networks are more likely to access diverse and non-redundant knowledge resources, thereby establishing the structural foundation for superior innovation outcomes. Conversely, firms located in closed and redundant network structures face significantly higher probabilities of falling into the low-innovation-performance group, especially when accompanied by weak digital transformation and unfavorable relational conditions.

Overall, this study demonstrates that firm innovation performance is the result of multiple structurally embedded and digitally conditioned paths, among which structural holes constitute the most fundamental driving force. By uncovering the configurational mechanisms through which network positions and digital transformation jointly influence innovation outcomes, the study provides a deeper understanding of the complex and non-linear nature of firm innovation.

### 5.2. Managerial Implications

The findings provide several actionable implications for manufacturing firms seeking to enhance innovation performance through the joint deployment of digital transformation and collaborative network strategies.

First, prioritizing structural hole positions to build non-redundant knowledge access channels. The dominant role of structural holes indicates that occupying brokerage positions in technological collaboration networks is the primary condition for achieving high innovation performance. For manufacturing firms, this means moving beyond stable and closed partnership structures and actively embedding themselves into heterogeneous innovation networks. In practice, firms should establish cross-regional and cross-industry R&D collaborations, participate in national innovation platforms and industrial technology alliances, connect with universities, research institutes, digital solution providers, and upstream-downstream firms simultaneously. Such strategies help firms bridge otherwise disconnected actors in the network, thereby gaining access to diverse technological knowledge and avoiding information redundancy. Compared with simply increasing the number of partners, occupying brokerage positions between different knowledge communities is more effective in improving innovation outcomes.

Second, implementing digital transformation as a network-enabled capability rather than a standalone investment. The results show that digital transformation does not automatically lead to high innovation performance; its effect depends on the firm’s network position. This implies that digital technologies should be deployed to enhance collaboration efficiency and knowledge integration rather than being treated as isolated infrastructure investments. Manufacturing firms should use digital platforms to manage inter-firm R&D collaboration, build data-sharing mechanisms with key innovation partners, apply AI and industrial internet technologies to integrate external knowledge into internal innovation processes. In other words, digital transformation should be embedded into the collaborative innovation process so that it amplifies the advantages brought by structural hole positions.

Third, adopting a configurational strategy instead of a single-factor optimization approach. The empirical results demonstrate that firm innovation performance is shaped by multiple equifinal paths rather than by the linear effect of any single antecedent condition, implying that manufacturing firms should shift from factor-oriented optimization logic to a configurational decision framework. In practical terms, this requires managers to evaluate digital transformation initiatives and network embedding strategies as interdependent elements within a coherent resource–structure system, rather than pursuing the maximization of individual indicators such as centrality or digital investment in isolation. For firms already occupying brokerage positions, the priority lies in leveraging digital technologies to accelerate heterogeneous knowledge recombination and cross-boundary collaboration, whereas firms embedded in dense and homogeneous networks need to first reconfigure their partnership portfolios to create non-redundant ties before scaling digital transformation efforts.

### 5.3. Limitations and Future Research

This study has some limitations that can serve as directions for future research. First, the measurement of collaborative network structure is based on co-patenting data among listed manufacturing firms, which primarily captures formal technological collaboration. Although this approach ensures data reliability and clear relational boundaries, it may overlook informal knowledge exchanges, supply-chain interactions, and platform-based digital collaborations that are increasingly important in the digital economy. Future studies could integrate multiple types of inter-firm relationships such as R&D alliances and industrial Internet platform participation to construct multiplex networks and examine how different layers of collaboration jointly influence innovation outcomes. Second, the digital transformation index is derived from textual analysis of annual reports using a domain-specific dictionary and TF-IDF weighting. While this method effectively reflects firms’ strategic attention to digital technologies, it mainly captures the cognitive and disclosure dimension of digitalization rather than its actual implementation depth or performance consequences. Subsequent research may combine textual indicators with objective measures, such as digital technology investment, software and data assets, or the adoption of intelligent manufacturing systems, to obtain a more comprehensive assessment of digital transformation.

## Figures and Tables

**Table 1:** Descriptive statistics and Pearson correlation results.

Variable	N	Mean	SD	Min	Max	*FIP*	*DT*	*DC*	*CC*	*SH*	*EC*
*FIP*	1292	22.00	21.930	0.000	138.1	1.000 ***					
*DT*	1292	0.001	0.001	0.000	0.015	0.265 ***	1.000 ***				
*DC*	1292	0.006	0.015	0.000	0.074	0.418 ***	0.105 ***	1.000 ***			
*CC*	1292	1.186	0.258	0.748	1.889	−0.008	0.03	0.213 ***	1.000 ***		
*SH*	1292	0.001	0.003	0.000	0.028	0.352 ***	0.092 ***	0.541 ***	0.283 ***	1.000 ***	
*EC*	1292	22.00	21.930	0.000	138.1	−0.064 *	0.047	0.153 ***	0.671 ***	0.224 ***	1.000 ***

Note: *** *p* < 0.001, ** *p* < 0.01, * *p* < 0.05.

**Table 2:** Predictive performance comparison of diverse models.

Model	Accuracy	Precision	Recall	AUC
Random Forest	0.669	0.706	0.635	0.709
Logistic	0.622	0.685	0.524	0.700
SVM	0.653	0.746	0.518	0.696
CART	0.659	0.667	0.706	0.718

**Table 3:** Key decision rules of *FIP*.

Conditional Factors					Criteria		Decision Factors
*DT*	*DC*	*CC*	*SH*	*EC*	*Support*	*Confidence*	*FIP*
≤33.853	-	≤0.038	≤1.063	-	47.16%	70.02%	Low
≤0.935	(0, 0.001]	≤0.038	≤1.063	-	1.55%	80.00%	High
(0.935, 33.853]	(0, 0.001]	≤0.038	≤1.063	-	13.52%	61.83%	Low
-	-	-	(1.063, 1.616]	≤0.008	31.48%	62.62%	High
-	-	-	(1.063, 1.616]	>0.008	2.06%	90.00%	Low
-	-	-	>1.616	-	8.36%	83.95%	High

**Table 4:** The regression analysis results of *FIP*.

	(1)	(2)	(3)	(4)
	*FIP*	*FIP*	*Logit* (*FIP*)	*Logit* (*FIP*)
*DC*	401.573 ***	275.782 ***	526.261 ***	560.307 ***
	(11.05)	(8.07)	(3.29)	(3.07)
*DT*	0.015 ***	0.016 ***	0.021 ***	0.025 ***
	(9.20)	(10.63)	(6.84)	(7.49)
*CC*	−4.893	−4.365	−4.552	−2.573
	(−1.48)	(−1.46)	(−0.83)	(−0.44)
*EC*	−72.571 ***	−38.756 **	−91.490 ***	−64.876 **
	(−4.22)	(−2.46)	(−2.96)	(−2.00)
*SH*	1.187 ***	0.669 ***	1.438 ***	0.670 *
	(7.10)	(4.30)	(4.20)	(1.75)
*Size*		0.425 ***		0.659 ***
		(12.53)		(8.60)
*Lev*		0.355		0.749 *
		(1.60)		(1.68)
*Board*		−0.253 *		−0.420
		(−1.71)		(−1.44)
*TobinQ*		0.065 **		0.298 ***
		(2.44)		(4.96)
*Roe*		1.282 ***		1.429 **
		(4.28)		(2.37)
*Firmage*		−0.158		0.243
		(−1.47)		(1.10)
*Constant*	1.292 ***	−6.775 ***	−2.457 ***	−16.966 ***
	(6.84)	(−9.26)	(−6.97)	(−10.07)
*N*	1292	1292	1292	1292

Note: * *p* < 0.10, ** *p* < 0.05, *** *p* < 0.01. *t* statistics in parentheses.

**Table 5:** Regression results with configurational condition derived from CART.

	(1)	(2)	(3)	(4)
	*FIP*	*FIP*	*Logit* (*FIP*)	*Logit* (*FIP*)
*Configuration1*	−0.497 ***	−0.457 ***	−0.858 ***	−0.897 ***
	(−4.09)	(−4.11)	(−4.14)	(−4.02)
*DC*	427.173 ***	302.866 ***	524.473 ***	543.506 ***
	(11.66)	(8.76)	(3.38)	(3.05)
*DT*	0.010 ***	0.012 ***	0.012 ***	0.016 ***
	(5.30)	(6.49)	(3.18)	(3.93)
*CC*	−7.978 **	−7.106 **	−9.420 *	−7.165
	(−2.37)	(−2.33)	(−1.71)	(−1.21)
*EC*	−63.242 ***	−31.031 **	−70.606 **	−45.515
	(−3.67)	(−1.97)	(−2.33)	(−1.42)
*SH*	0.521 **	0.055	0.286	−0.487
	(2.24)	(0.26)	(0.65)	(−1.02)
*Size*		0.418 ***		0.660 ***
		(12.41)		(8.49)
*Lev*		0.395 *		0.811 *
		(1.79)		(1.79)
*Board*		−0.328 **		−0.579 *
		(−2.21)		(−1.93)
*TobinQ*		0.062 **		0.296 ***
		(2.34)		(4.88)
*Roe*		1.314 ***		1.484 **
		(4.41)		(2.44)
*Firmage*		−0.127		0.289
		(−1.19)		(1.30)
*Constant*	2.409 ***	−5.555 ***	−0.491	−14.790 ***
	(7.27)	(−7.07)	(−0.84)	(−8.31)
*N*	1292	1292	1292	1292

Note: * *p* < 0.10, ** *p* < 0.05, *** *p* < 0.01. *t* statistics in parentheses.

## Data Availability

Data in this study are available from the corresponding author upon reasonable request.
